# Comparison of radiotherapy techniques with flattening filter and flattening filter-free in lung radiotherapy according to the treatment volume size

**DOI:** 10.1038/s41598-020-66079-6

**Published:** 2020-06-02

**Authors:** Alaettin Arslan, Burak Sengul

**Affiliations:** 1 Kayseri City Hospital, Turkey, Clinic of Radiation Oncology, Doctor, Kayseri, Turkey; 2 Kayseri City Hospital, Turkey, Clinic of Radiation Oncology, Health Physicist, Kayseri, Turkey

**Keywords:** Cancer, Oncology, Physics

## Abstract

In external radiotherapy (RT), the use of flattening filter-free (FFF) radiation beams obtained by removing the flattening filter (FF) in standard linear accelerators is rapidly increasing, and the benefits of clinical use are the issue of research. Advanced treatment techniques have increased the interest in the operation of linear accelerators in FFF mode. The differences of the beams with non-uniform dose distribution created by removing FF compared to the beams with uniform dose distribution used as a standard were examined. These differences were compared in the treatment plans of lung patients who have different planning target volumes (PTV). Clinac IX linear accelerator units were used. Twenty patients with previously completed treatment were divided into two groups depending on the size of the target volume. All patients underwent two different intensity-modulated RT (IMRT) plans using FF and FFF beams. The Wilcoxon Signed-Rank test was used to compare two different techniques (Significance p < 0.05). There was no statistically significant difference between the two techniques when looking at the D2%(Gy), D98%(Gy), D50%(Gy), homogeneity (HI), and conformity index (CI) data for both groups. When the critical organ doses were evaluated, there was a statistically significant difference only in the V20(%) values of the lungs, but these differences were not very large. Monitor unit (MU) data were found to be lower in FF planning, and treatment time was lower in FFF planning. Except for shorter treatment times, and of the lungs V20(%) value, in standard fractionated RT of lung cancer, there was no significant difference between the use of FFF and FF techniques for large and small target volumes.

## Introduction

Although the use of flattening filter free (FFF) radiation beams obtained by lifting flattening filter (FF) in standard linear accelerators is increasing rapidly in RT, the benefits of clinical use are researched. A linear accelerator with the FF removed produces an irregular dose profile beam^1^. Initially, the removal of the FF was performed manually. The most significant benefit of FFF irradiation for IMRT is thought to be that increased dose rate with reduced head scattering and leakage radiation leads to improved dose calculation and provides a more uncomplicated, faster, and more accurate dose to normal tissues with reduced dose^2^. In a study by Vassiliev ON^3^ *et al*., it has been shown that better radiation treatments can be improved with an accelerator that does not include a FF.

FFF beams provide a more intense X-ray beam at the center than conventional FF photon rays. The high dose rate provided by the FFF beams reduces beam duration and increases clinical efficiency^4,5^. The possible reason is that the attenuation effect of radiation is reduced in FFF mode. At greater distances, the out-of-field dose was decreased because of reduced head leakage^6^. Advanced treatment techniques such as stereotactic RT or intensity-modulated RT (IMRT) have increased interest in the operation of linear accelerators in FFF mode. Dosimetric properties of FFF rays have an impact on treatment delivery, patient comfort, dose calculation accuracy, beam matching, absorbed dose detection, treatment planning, machine-specific quality assurance, imaging, and radiation protection^7^. Javedan K^8^ *et al*., investigated the superficial dose of conventional FF beam and FFF beam using the Monte Carlo method. As a result, the Monte Carlo simulation showed that the surface dose was higher compared to the FF beam due to low average energy in the FFF beam.

In this study, the differences of the beams with non-uniform dose distribution were compared with the beams with uniform dose distribution as standard. These differences were compared in the treatment plans of 20 lung patients with different target volumes (PTV). The primary purpose of the comparison is to investigate the characteristics of the FFF beams, where low-dose regions occur when moving from the center to the edges, and what the benefits of large and small target volume plans will be. The patients were divided into two groups according to PTV volumes as A (PTV < 500 cc) and B group (PTV > 1000 cc). This comparison was based on statistical differences of monitor unit (MU) values, critical organ doses, and PTV evaluation criteria in treatment plans.

## Material and method

### Patient selection and planning

In our study, Clinac IX linear accelerator units (the Eclipse 15.1 version treatment planning system) developed by Varian Medical System (Palo Alto, CA USA) was used. Twenty-eight patients who had previously completed treatment were evaluated. Two planning computed tomographies (CT), with and without contrast, were obtained. Target volumes were re-contoured on contrast-enhanced CT and then transferred to non-contrast CT. Critical organs were then identified and contoured. Initially, 28 patients were discussed. After the target volume measurements, 8 patients with ≥500 and ≤1000 cc PTV volumes were excluded from the study. Because the main purpose of the study was to compare patients with high target volume differences. The remaining 20 patients were included in the study with target volumes below 500 cc (n = 10, group A) and above 1000 cc (n = 10, group B). The mean treatment volume of the patients in group A was 214.61cc (69.1cc-388cc), and the treatment volume of group B was 1516.6cc (1044cc-3030cc).

All patients underwent two separate IMRT plans using FF and FFF beams. In the planning, treatment fields between 5–9 were used using 6 MV photon energies. A total dose of 60 Gy was defined from 2 Gy per fraction. 95% of the target volumes (D95%) were intended to receive a 60 Gy treatment dose, with a dose homogeneity of 95–107%. The dose rate was selected as 400 MU/min for FF plans, and 1400 MU/min for FFF planning. In the planning techniques for the critical organs entering the target volumes, the remaining critical organ volumes were created by subtracting the target volumes with a 2 mm margin to prevent the dose homogeneity in the target volumes. Before the optimization, ring structures were formed around the target volume to prevent the formation of treatment doses outside the target volumes, and dose restrictions were applied to these structures during optimization. Photon Optimizer (PO) optimization algorithm and intermediate-dose calculation were used as the optimization algorithm. Value functions for target volumes were defined as the top priority for value definition during optimization. For the critical organs, it was prioritized according to the relationship with the target volume, and the optimization process was repeated until an optimal treatment plan was obtained. The dose limits for the critical organs in the optimization process are shown in Table 1. Optimization goals and iteration numbers were kept the same for both techniques. Analytical Anisotropic Algorithm (AAA) was used for multileaf collimator movements and dose calculations of obtained beam flux.

**Table 1:** Critical organ dose limitations.

Spinal Cord	Dmaximum (Gy)	<45 Gy
Lung	V20 (%)	<%35
	V5(%)	<%65
	Dmean (Gy)	<20 Gy
Heart	V40 (%)	<%80
	Dmean (Gy)	<26 Gy
Esophagus	Dmean (Gy)	<34 Gy

In the evaluation of treatment volumes, the near-maximum (D2%) dose, the average dose (D 50%), and the near-minimum (D98%) dose defined in the protocol of ICRU 83 Report were examined^9^. For homogeneity index *(HI of zero is ideal; (D2%-D98%)/D50%)* and for conformity index *(CI of 1.0 is ideal; Volume of PTV covered by the 95% isodose curve/Volume of PTV)* formulas were used^9,10^.

### Statistical analysis

IBM SPSS version 24.0 (SPSS Inc., IL, USA) was applied for statistical comparison. Conformity of variables to normal distribution was evaluated by visual, and analysis methods, and non-parametric tests were used since it was observed that it did not fit the normal distribution. The Wilcoxon Signed-Rank test, which is a nonparametric binary comparison test, was used to compare two different techniques (Significance p < 0.05). The mean and standard deviation values of the data were chosen as terms defining the differences between the techniques.

The study was conducted with the approval of the Non-Interventional Clinical Research Ethics Committee of Erciyes University Faculty of Medicine. Ethics Committee convened on 13.11.2019 and received the protocol number 2019/775.

### Ethical statement

All procedures performed in studies involving human participants were in accordance with the ethical standards of the institutional and/or national research committee and with the 1964 Helsinki Declaration and its later amendments or comparable ethical standards.

### Informed consent

The informed consent form was obtained from all subjects. Institutional review board approval was obtained for this study. The study was conducted with the approval of the Non-Interventional Clinical Research Ethics Committee of Erciyes University Faculty of Medicine. Ethics Committee convened on 13.11.2019 and received the protocol number 2019/775.

## Results

Table 1 shows the critical organ dose limitations that are referenced when planning. The maximum dose to the spinal cord, average dose to the esophagus, the V40(%), and average dose to the heart, the V5(%), V20(%), and average dose to the lungs have been taken into consideration.

The mean ± standard deviation and significance values of the data obtained from IMRT-FF and IMRT-FFF plans for groups A and B are shown in Tables 2 and 3, respectively.

**Table 2:** FF/FFF plan comparison in group A patients.

PTV < 500cc		6MV IMRT-FF		6MV IMRT-FFF		p-value
Grup A		Mean	St. Dev.	Mean	St. Dev.	
PTV	D%2 (Gy)	62,61	±0,73	62,63	±0,66	0,646
	D%98(Gy)	59,26	±0,35	59,22	±0,33	0,44
	D%50 (Gy)	61,45	±0,51	61,46	±0,44	0,721
HI		0,054	±0,014	0,055	±0,013	0,285
CI		1,11	±0,13	1,12	±0,13	0,515
Spinal Cord	Dmax(Gy)	30,92	±9,05	30,16	±10,32	0,066
Lung	V20 (%)	18,43	±9,03	17,87	±8,85	**0,005**
	V5 (%)	46,35	±11,07	45,21	±10,51	0,093
	Dmean(Gy)	10,88	±3,38	10,62	±3,21	0,09
Heart	V40 (%)	3,21	±3,83	3,01	±3,64	0,18
	Dmean(Gy)	7,05	±5,51	6,93	±5,54	0,114
Esophagus	Dmean(Gy)	18,22	±6,84	18,08	±6,90	0,126
Normal tissue	V5Gy (%)	23,33	±9,53	22,53	±9,18	0,12
Monitor Unit		869	±199	1018	±221	**0,005**

PTV: Planning target volume.

IMRT-FF: Intensity-modulated RT-flattening filter.

IMRT-FFF: Intensity-modulated RT-flattening filter free.

HI: Homogeneity index.

CI: Conformity index.

MU: Monitor unit.

**Table 3:** FF/FFF plan comparison in group B patients.

PTV > 1000cc		6MV IMRT-FF		6MV IMRT-FFF		p-value
Grup B		Mean	St. Dev.	Mean	St. Dev.	
PTV	D%2 (Gy)	63,12	±0,71	63,16	±0,70	0,413
	D%98(Gy)	58,82	±0,53	58,73	±0,33	0,139
	D%50 (Gy)	61,85	±0,62	61,89	±0,63	0,76
HI		0,069	±0,01	0,072	±0,01	0,285
CI		1,03	±0,04	1,04	±0,04	0,241
Spinal Cord	Dmax(Gy)	37,76	±4,04	37,73	±4,37	0,799
Lung	V20 (%)	29,65	±5,91	28,81	±5,74	**0,005**
	V5 (%)	76,09	±16,53	75,92	±16,8	0,241
	Dmean(Gy)	17,63	±2,77	17,48	±2,73	0,093
Heart	V40 (%)	16,34	±9,31	16,22	±8,75	0,26
	Dmean(Gy)	20,47	±8,23	20,31	±8,79	0,307
Esophagus	Dmean(Gy)	24,41	±5,12	24,31	±5,12	0,386
Normal tissue	V5Gy (%)	49,99	±14,80	49,49	±15,12	0,169
Monitor Unit		1443	±368	1731	±489	**0,007**

PTV: Planning target volume.

IMRT-FF: Intensity-modulated RT-flattening filter.

IMRT-FFF: Intensity-modulated RT-flattening filter free.

HI: Homogeneity index.

CI: Conformity index.

MU: Monitor unit.

**Figure 1:**
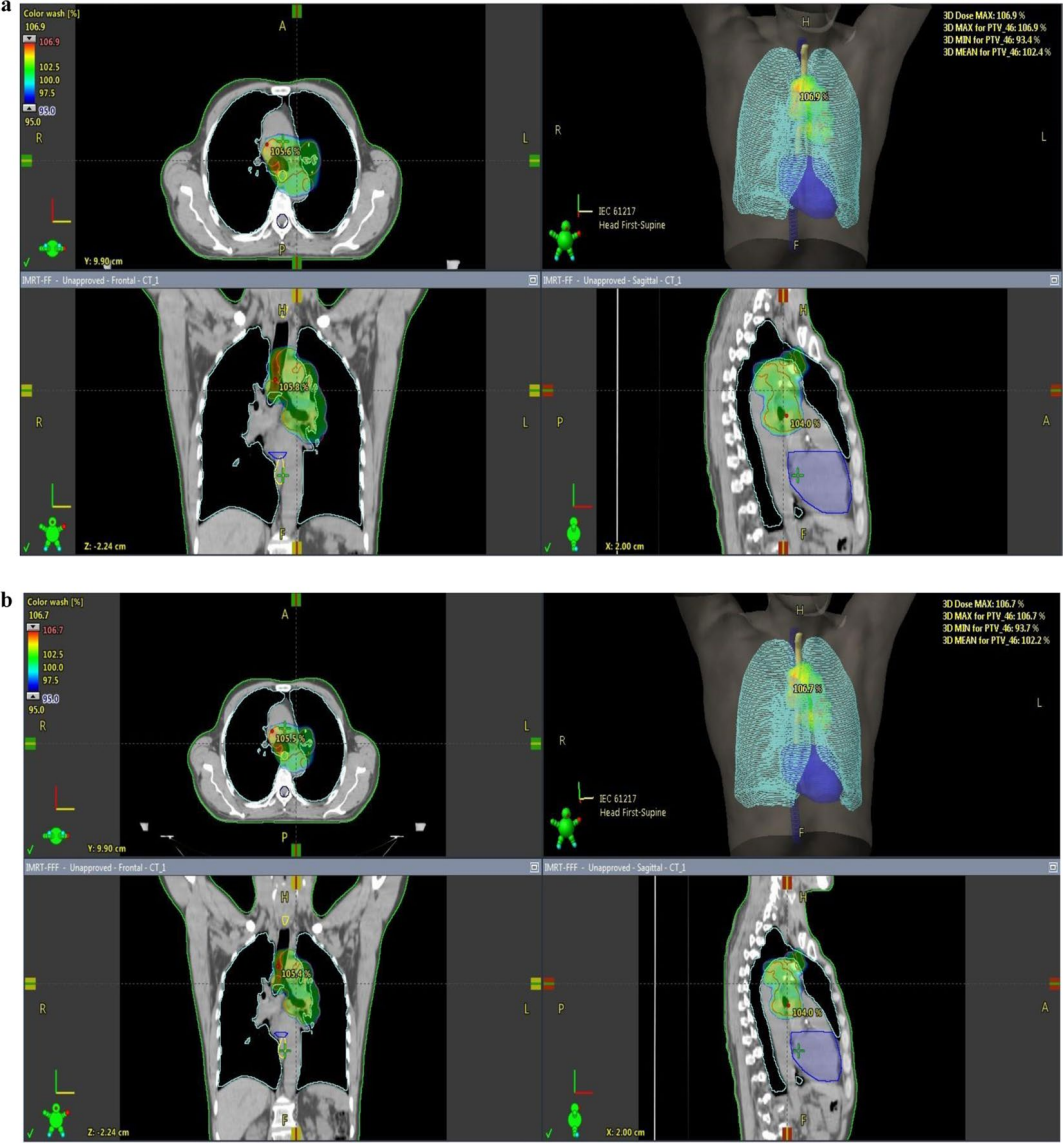
(**a**) FF planning of a patient in group A. [Patient with small target volume, 95% of the target volumes (D95%) were intended to receive a 60 Gy treatment dose, with a dose homogeneity of 95–107%.] **(b)** FFF planning of a patient in group A. [Patient with small target volume, 95% of the target volumes (D95%) were intended to receive a 60 Gy treatment dose, with a dose homogeneity of 95–107%.].

**Figure 2:**
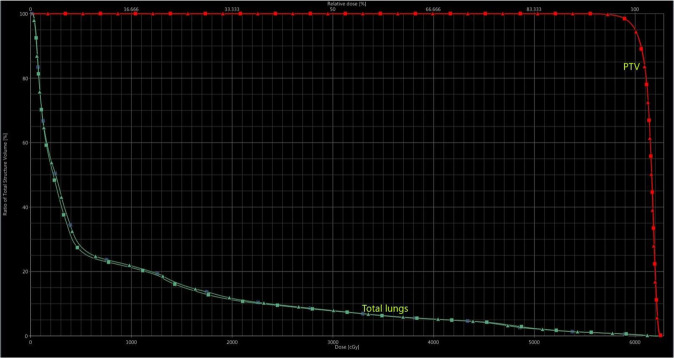
Dose-volume histogram of a patient in group A. [In the histogram, PTV and total lung dose curves are shown according to FF and FFF planning techniques in a patient with a target volume of 74.8 cc. Lung V20(%) appears to be lower in FFF planning. *PTV: Planning target volüme, FF: Flattening filter* (►), *FFF: Flattening filter-free* (■)].

Figure 1 shows the dose distributions of FF and FFF planning of a patient in group A. In group A (PTV < 500 cc), the values of PTV D2%(Gy), D98%(Gy), D50%(Gy) were similar between FF and FFF, and there was no statistically significant difference. Again, HI and CI values were similar, and there was no statistical difference. When the critical organ doses were examined in group A; lung volume receiving 20 Gy dose (V20); it was found 18.43% in FF irradiation and 17.87% in FFF irradiation. FFF irradiation was more reliable than FF irradiation, and there was a statistically significant difference (p = 0.005) **(**Fig. 2**)**. There was no difference in the volume of the lung (V5(%)) and the mean lung dose. Other critical organs, heart (V40% and mean), esophagus, spinal cord, and body volume receiving 5 Gy dose (V5%), were not statistically different. The mean value of MU was 869 in FF irradiation and 1018 in FFF irradiation, and FF was found to be statistically significant compared to FFF (p = 0.005). The mean Beam on Time (BoT) value was 2.17 for FF irradiation and 0.72 minutes for FFF irradiation.

**Figure 3:**
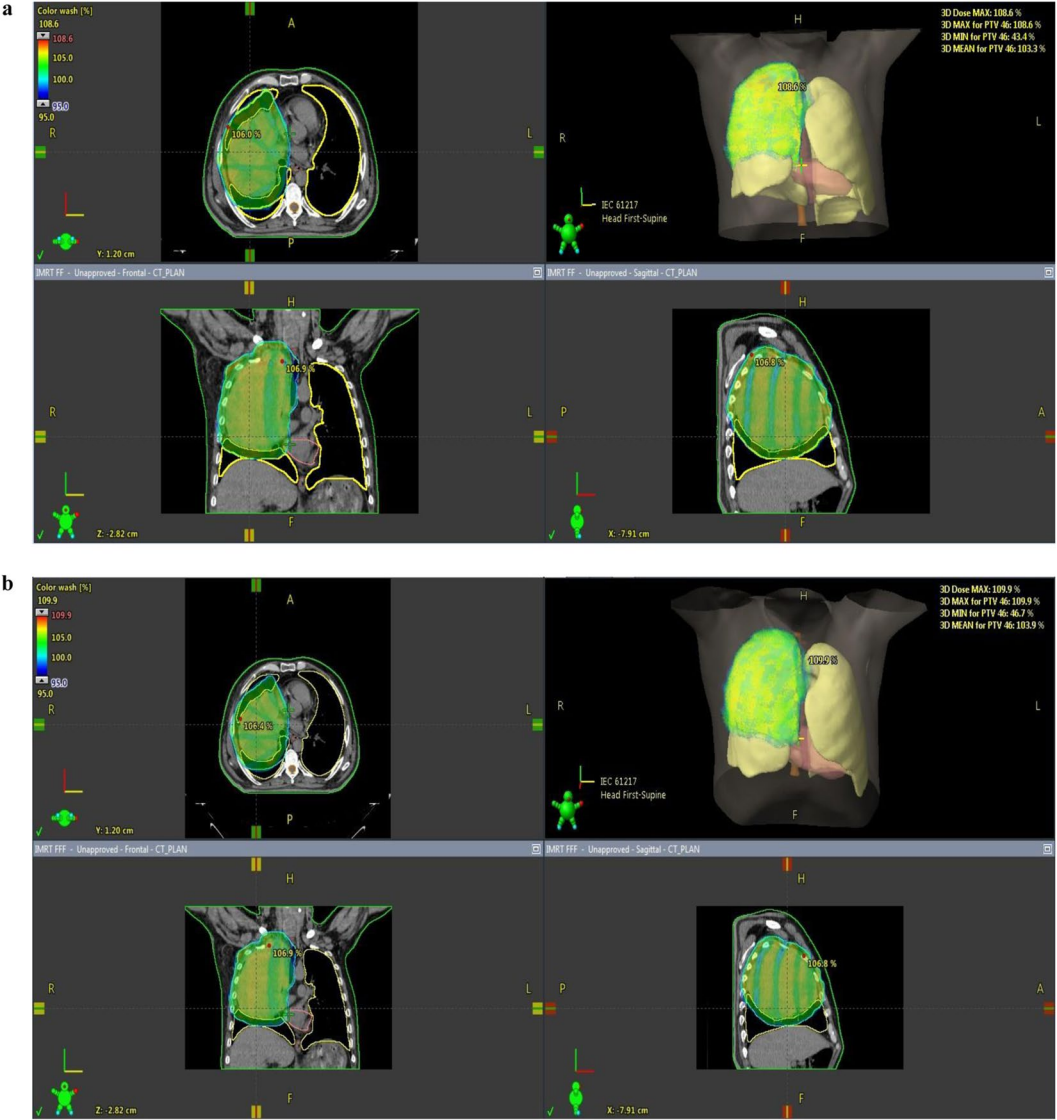
(**a**) FF planning of a patient in group B. [Patient with large target volume, 95% of the target volumes (D95%) were intended to receive a 60 Gy treatment dose, with a dose homogeneity of 95–107%.] **(b)**. FFF planning of a patient in group B. [Patient with large target volume, 95% of the target volumes (D95%) were intended to receive a 60 Gy treatment dose, with a dose homogeneity of 95–107%.].

**Figure 4:**
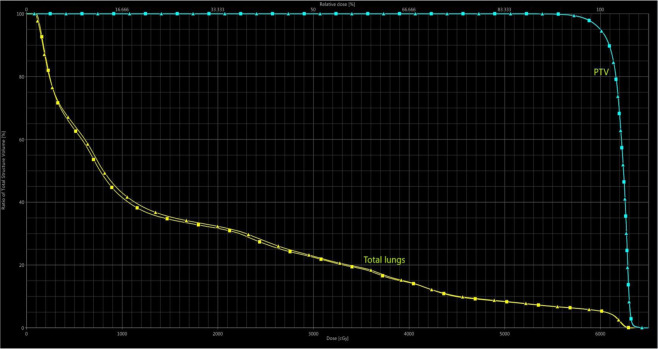
Dose-volume histogram of a patient in group B. [In the histogram, PTV and total lung dose curves are shown according to FF and FFF planning techniques in a patient with a target volume of 1044.4 cc. Lung V20(%) appears to be lower in FFF planning. *PTV: Planning target volüme, FF: Flattening filter (*►*), FFF: Flattening filter-free* (■)].

Figure 3 shows the dose distributions of FF and FFF planning of a patient in group B. In group B (PTV > 1000 cc), the values of PTV D2%(Gy), D98%(Gy), D50%(Gy) were similar between FF and FFF, and there was no statistically significant difference. HI, and CI values were similar, and there was no statistically significant difference. When the critical organ doses were examined in group B; lung volume receiving 20 Gy dose (V20(%)); it was found that 29.65% in FF irradiation and 28.81% in FFF irradiation. FFF irradiation was more reliable than FF irradiation, and there was a statistically significant difference (p = 0.005) **(**Fig. 4**)**. There was no statistically significant difference between the mean lung dose and the lung volume (V5%) receiving 5 Gy dose. There was no statistically significant difference in body volume receiving 5 Gy dose (V5%), heart (V40% and mean), esophagus, and spinal cord from other critical organs. The value of MU was 1443 in FF irradiation and 1731 in FFF irradiation, and FF was statistically significant compared to FFF (p = 0.007). The mean BoT value was 3.60 for FF irradiation and 1.23 minutes for FFF irradiation.

When A and B groups were compared, critical organ doses were naturally higher due to the large treatment volume of patients in group B. This is particularly evident in the body volume receiving 5 Gy dose and in the heart (V40% and average dose).

## Discussion

In the literature, many studies are showing the advantages of FFF irradiation over FF irradiation^11–16^, and FFF is highly used in our clinic as in many centers. Especially with advancing technology and new RT devices, many studies are being done in this direction. In our study, the clinical advantages and disadvantages of treatment plans obtained by using FFF beams, depending on the size of the target volume in the lung planning, against treatment plans obtained with FF beams were examined. In the literature, there are not many studies examining FF and FFF techniques, depending on the size of the target volume in the treatment of lung cancer.

As seen in our study, one of the important differences between FF and FFF is the duration of dosing. In a study where the effect of BoT differences based on the microdosymmetric kinetic model (MCM) was investigated, the effect of dosing time with FF and FFF beams was investigated. It was pointed out that dosing times were shortened in the FFF technique, and errors decreased both in clinical efficiency and during fractions. Furthermore, it has been shown that the dose administration time affects the relative biological effectiveness (RBE) during photon irradiation. As a result, it was stated that the effects of dosing time are important and should be considered in radiation therapy^17^.

In a study on cervical cancer, 6 MV photon has compared with FF and FFF beams in IMRT technique. In stage 2-3B patients, 50,4 Gy RT was planned in 28 fractions, and there was a statistically significant difference in HI, CI, bladder V50Gy, MU, D50%, and D2% PTV doses and non-tumor low dose volumes. At the end of the study, it was stated that FFF irradiation has the advantage of providing faster treatment to normal tissues with fewer doses. It has been pointed out that the selection of advanced innovative technologies will play an important role in modern RT and increase patient safety, reduce patient waiting time, and reduce the chance of developing secondary cancer after RT^18^.

In the study of Youqun Lai^19^ *et al*., the VMAT technique was used, and HI and dose distribution were superior to in the FFF irradiation to FF. Also, the average BoT value was found to be 42,8% lower. In the study of Lu^20^ *et al*., evaluating sinonasal cancers, better contralateral optic dose values were obtained in FFF beams and IMRT plans, while comparable results were observed in VMAT plans. Brendan M^5^ and colleagues in the study of lung and liver SBRT for the technique, FFF irradiation compared to a conventional FF, treatment and immobilization time, was observed to reduce by close to 50%. Thu M Dang^1^ and colleagues in their study to measure the effectiveness of FFF irradiation for SBRT did not find significant differences in CI and HI values, but gradient index (GI) found a significant favor of FFF irradiation. They recommended FFF irradiation in SBRT because it was faster, reduced organ movement during fraction, patient retention time, and overall treatment time.

In a study of non-small cell stage I lung cancer, the use of FFF rays for stereotactic radiation therapy significantly reduced the qualitatively comparable dose distributions with the flattening rays and the treatment time. The use of the X6FFF beam increased the appropriateness of the dose distribution, while the X10FFF beam offered a slight improvement in treatment efficiency and lower skin and peripheral dose. However, all effects were considered relatively minor^21^. In another study on lung cancer, 25 patients scheduled for SBRT were presented with a 24 Gy dose of 6 MV FFF VMAT in a single fraction, and these treatment plans were compared to FF VMAT. No statistically significant difference was found in dosimetric results and MU between FF and FFF treatments. On the other hand, while FFF VMAT provides equivalent dosimetric results to target volume and organs at risk, it significantly reduced the duration of treatment compared to FF VMAT^22^.

Although FFF radiation has been in the clinic for some time, it has been used in certain devices in small volume tumors. Indeed, TomoTherapy machines do not have a FFF, and the first treatments with that technology were delivered in around. Recently it has been used in linear accelerators with 6 and 10 MV X-rays. At the large irradiation volumes, the intensity of the beam at the central point must be modulated by MLC motions of the FFF X-ray beam. Therefore, higher MU values are required compared to conventional X-rays. In other words, MLC motions (IMRT) are used to “flatten” FFF X-rays to provide dose homogeneity in large PTVs. High dose rates from FFF X-rays are offset by greater MU requirements^23^. In a study on breast RT, FF and FFF irradiation were compared on the large target volume. No significant differences were observed in terms of PTV coverage, homogeneity, or suitability. In addition, there was no statistically significant difference between PTV size and plan quality. There was a 31% decrease in BoT values in favor of FFF, but this decrease led to a 10% reduction in the total treatment time^24^. Depending on the size of the PTV (1,52 cm^3^ to 445,24 cm^3^), in the study in which FFF beams were compared in a dynamic conformal arc (DCA) or Rapid Arc (RA) technique; FFF rays provided better protection of healthy tissues, except for 10FFF used with DCA. 6FFF was found to be slightly better than 10FFF in terms of healthy tissue average doses, and it was stated that 10FFF used with DCA should be used carefully for medium and large volumes. In the context of this study, it seems that FFF-DCA will be preferred for small volumes (<20 cm^3^); FFF-DCA or FFF-RA for 20 cm^3^ <PTV < 50 cm^3^ and FFF-RA for medium (>50 cm^3^) and large volumes (>100 cm^3^)^25^.

Our study aimed to investigate the difference between FFF irradiation and FF irradiation, in dose distributions and critical organ doses due to differences in the treatment volume. There was no significant difference except lung V20(%) volume, but MU and BoT values were in parallel with the literature. This dosimetric advantage should also be supported by clinical studies, and there is a research planned by us on the subject.

## Conclusion

Removing the filter from a standard linear accelerator result in an increase in dose rate, reduced scattering from the filter, a decrease in beam intensity from the center of the beam field to the edges, and a non-uniform beam profile. It has been investigated what differences these changes will bring in the irradiation of large and small treatment volumes.

As a result, when the critical organ doses were evaluated, there was a statistically significant difference only in the V20(%) values of the lungs, but these differences were not very large. When MU values were compared, it was seen that the values in the IMRT-FFF technique were higher than those in the IMRT-FF technique. The higher MU values of the FFF treatment plans are thought to be due to the difference in the beam profile. The BoT value was low in FFF planning in both groups, which seems to be important in reducing device density and errors caused by patient movements. This is a dosimetric study, and further studies are needed for both lung and other cancer sites.
